# Supplementary material for: Generation and Characterisation of Monoclonal Antibodies against Nairobi Sheep Disease Virus Nucleoprotein
Source: Viruses. 2023 Sep 5;15(9):1876. doi: 10.3390/v15091876 (PMC10536980; doi:10.3390/v15091876)
Supplement: Supplementary file 1 [file viruses-15-01876-s001.zip › viruses-2559551-supplementary.pdf]

Brief Report

Generation and characterization of monoclonal antibodies against Nairobi sheep disease virus nucleoprotein

Emmanuel A. Maze[1*]; Tiphany Chrun[1‡]; George Booth[1‡]; Georgina Limon[1*]; Bryan Charleston[1†]; Teresa Lambe[2†]

[1] The Pirbright Institute, Ash Road, Pirbright, Woking, GU24 0NF, United Kingdom; chrun.tiphany@gmail.com (T.C.); gb5g19@soton.ac.uk (G.B.); georgina.limon-vega@pirbright.ac.uk (G.L.); bryan.charleston@pirbright.ac.uk (B.C)

[2] Oxford Vaccine Group, Centre for Clinical Vaccinology and Tropical Medicine (CCVTM), Churchill Hospital Old Road, Headington, Oxford, OX3 7LE, United Kingdom; teresa.lambe@paediatrics.ox.ac.uk (T.L.)

‡† Contributed equally

* Correspondence: emmanuelatanganamaze@gmail.com (E.A.M.); georgina.limon-vega@pirbright.ac.uk (G.L.)

Supplementary materials

Table S1. Genbank accession numbers of viruses used for the alignment in Figure 1. NSDV: Nairobi sheep disease virus; DUGV: Dugbe virus; CCHFV: Crimean-Congo haemorrhagic fever virus.

Virus name	Accession number	Strain/Isolate	Region/Country of origin
AAM33324.1 NSDV G619	AAM33324.1	Ganjam G619	India
AED88230.1 NSDV 779	AED88230.1	779	India
YP_009361831.1 NSDV Jilin	YP_009361831.1	Jilin	China
AYI99259.1 NSDV Hubei	AYI99259.1	Hubei	China
AAM33323.1 NSDV 708	AAM33323.1	708	Kenya
AAL73399.1 DUGV IbAr 1792	AAL73399.1	Ibar 1792	Nigeria
ABY82500.1 Kupe virus K611	ABY82500.1	K611	Kenya
BAU51653.1 Tofla virus Nag-Hfor-2014	BAU51653.1	Nag-Hfor-2014	Japan
AAA43842.1 HAZV JC280	AAA43842.1	JC280	Pakistan
AAK32973.1 CCHFV 88166	AAK32973.1	88166	China

ASZ84923.1 CCHFV Russia_Kalmykia_Shch_2_2016	ASZ84923.1	Russia_Kalmykia_Shch_2_2016	Russia
ABA18804.1 CCHFV Congo 3010	ABA18804.1	Congo 3010	Democratic Republic of the Congo
YP_009513193.1 Thiafora virus AnD 11411	YP_009513193.1	AnD 11411	Senegal
AFH89034.1 Erve virus JF911699.1	AFH89034.1	-	France
AMT75433.1 Tillamook virus RML 86	AMT75433.1	RML 86	USA
AMR73397.1 Taggert virus KT820207.1	AMR73397.1	MI14850	Australia
AMT75421.1 Sakhalin virus LEIV-71S	AMT75421.1	LEIV-71C	Russia
AMT75388.1 Clo Mor virus ScotAr7	AMT75388.1	SCOT Ar7	Scotland
YP_009551598.1 Avalon virus CanAr173	YP_009551598.1	CanAr173	Canada
AJG39317.1 Wenzhou Tick Virus TS1-2	AJG39317.1	TS1-2	China
YP_009304988.1 Tacheng Tick Virus TC253	YP_009304988.1	TC253	China
YP_009293589.1 Huangpi Tick Virus H124-1	YP_009293589.1	H124-1	China
AKC89321.1 Qalyub virus EgAr 370	AKC89321.1	EgAr 370	Egypt
AMT75385.1 Bandia virus RV611	AMT75385.1	RV611	Senegal
AKC89315.1 Uzun Agach virus LEIV-Kaz155	AKC89315.1	LEIV-Kaz155	Kazakhstan
YP_009361834.1 Keterrah virus P61361	YP_009361834.1	P61361	Malaysia
ALD84348.1 Issyk-Kul virus LEZ 86-787	ALD84348.1	LEZ 86-787	Germany

ALD83624.1 Gossas virus DakAnD 401	ALD83624.1	DakAnD 401	Senegal
ALD84360.1 Yogue virus DakAnD 56	ALD84360.1	DakAnD 56	Senegal
YP_009111286.1 Leopards Hill virus 11SB17	YP_009111286.1	11SB17	Zambia
YP_009246487.1 Kasokero virus isolate Z-52963	YP_009246487.1	Z-52963	Uganda
KU343165.1 TRANSLATED Sapphire II virus 52301-14	KU343165.1	52301-14	USA
AMT75391.1 Dera Ghazi Khan virus JD154	AMT75391.1	JD154	Pakistan
AMT75376.1 Abu Mina virus EG AN 4996	AMT75376.1	EG AN 4996	Egypt
AMT75373.1 Abu Hammad virus Art 1194	AMT75373.1	Art 1194	Egypt
YP_009551666.1 Zirqa virus Por 7866	YP_009551666.1	Por 7866	United Arab Emirates
AMT75427.1 Soldado virus TRVL 52214	AMT75427.1	TRVL 52214	Trinidad and Tobago
AMT75418.1 Raza virus 829	AMT75418.1	829	Mexico
AMT75412.1 Punta Salinas virus CalArt888	AMT75412.1	CalArt888	Peru
AMT75409.1 Hughes virus G2126	AMT75409.1	G2126	USA
YP_009551659.1 Great Saltee virus RML 59972-6	YP_009551659.1	RML 59972-6	Ireland
AMT75400.1 Farallon virus CalfAr846	AMT75400.1	CalfAr846	USA
AKC89348.1 Caspiy virus LEIV-63Az	AKC89348.1	LEIV-63Az	Azerbaijan

Table S2. Range of sequence homology per species to NSDV. Homology is provided as the percentage of matching identity to NSDV G619 (AAM33324.1) estimated based on alignment in Figure 1, after removing the gaps manually.

	Percentage (%) of identity per species for each peptide	
Species	Peptide SG…SES (#1)	Peptide PR…NAH (#2)
Nairobi sheep disease nairovirus	93-100	86-100
Dugbe nairovirus	21-36	29-33
Hazara nairovirus	21-29	24
Crimean-Congo haemorrhagic fever nairovirus	7	19
Thiafora nairovirus	29-36	14
Sakhalin nairovus	14-21	5-14
Burana nairovirus	0	10-24
Qalyub nairovirus	21	10-19
Ketarah nairovirus	7-21	5-10
Kasokero nairovirus	21-36	19-24
Dera Ghazi Khan nairovirus	7	0-19
Hughes nairovirus	0-14	0-14

Table 3. Binding of monoclonal antibodies from hybridoma cultures to NSDV-derived peptide by ELISA, and NSDV-infected cells by IFA. O.D. values are provided for ELISA. IFA results are presented as the percentage (%) of fluorescent area to total area (confluence) occupied by cells in Incucyte images. ND = not done.

	List of Hybridomas	ELISA (O.D. values)		IFA (% positive)	
		PR…NAH (#2)	SG…SES (#1)	NSDV (+)	NSDV (-)
M1	AA2	0.06	1.42	0.15	0.02
	AC6	2.31	0.13	47.43	31.52
	AC11	1.98	0.09	0.24	0.13
	AD4	0.05	1.53	0.02	0.04
	AE2	0.05	1.99	45.73	0.02
	AE8	0.05	1.33	0.07	0.05
	AF5	0.05	2.51	82.22	0.08
	BC4	0.05	1.77	0.05	0.01
	BC6	0.06	1.27	0.05	0.02
	BF5	0.06	1.79	0.08	0.04
	BG2	0.06	1.75	0.03	0.05
	CB9	0.08	1.59	0.06	0.17
	CG2	0.06	1.55	0.10	0.07
	CG7	0.05	2.61	0.09	0.01
	CH7	0.06	1.83	0.51	0.04
	EA2	0.08	1.76	0.02	0.01
	FB6	0.05	1.54	0.04	0.03
	GA7	0.06	1.91	0.03	0.08
	GB12	0.06	1.34	0.02	0.00
	GC2	0.06	1.37	0.02	0.01
	GF2	2.19	0.09	0.03	0.02
	GH6	0.06	2.13	0.03	0.01
	GG12	0.06	2.16	61.39	0.06
	HD3	1.66	0.12	0.02	0.02
	HF3	0.06	1.43	0.03	0.02
	HH12	0.06	1.69	0.03	0.01
	IC8	0.06	1.34	0.03	0.01
M2	AA5	2.54	0.08	0.55	0.05
	AA6	0.74	0.13	ND	ND

	AH9	0.05	1.85	0.04	0.03
	CG6	0.05	2.25	0.11	0.05
	EA5	0.05	2.57	0.10	0.01
	EC3	0.06	2.00	0.02	0.05
	EE9	0.26	0.07	ND	ND
	EF11	2.76	0.09	0.02	0.03
	EG6	1.32	0.14	7.96	10.07
	FB10	3.00	2.67	43.02	10.46
	FC6	0.05	2.02	0.03	0.04
	FD8	0.05	1.93	0.03	0.02
	FE10	0.30	0.18	ND	ND
	FH3	2.45	2.48	24.53	1.84
	GF5	2.32	0.08	0.14	0.02
	GH10	0.06	0.07	ND	ND
	HB11	2.70	0.08	0.02	0.01
	IC8	2.82	0.13	0.10	0.01
	JE10	0.06	2.02	0.02	0.02
